# Bis[(*E*)-4-bromo-2-(methoxy­imino­meth­yl)phenolato-κ^2^ *N*,*O* ^1^]copper(II)

**DOI:** 10.1107/S1600536809047989

**Published:** 2009-11-18

**Authors:** Lan-Qin Chai, Jian Yao, Shang-Sheng Gong

**Affiliations:** a School of Chemical and Biological Engineering, Lanzhou Jiaotong University, Lanzhou 730070, People’s Republic of China

## Abstract

In the title centrosymmetric mononuclear copper(II) complex, [Cu(C_8_H_7_BrNO_2_)_2_], the Cu^II^ atom, lying on an inversion centre, is four-coordinated in a *trans*-CuN_2_O_2_ square-planar geometry by two phenolate O atoms and two oxime N atoms from two symmetry-related *N*,*O*-bidentate oxime-type ligands. Inter­molecular C—H⋯O hydrogen bonds link neighbouring mol­ecules into a one-dimensional supra­molecular structure with an *R*_2_^2^(14) ring motif. This structure is further stabilized by π–π stacking inter­actions between adjacent benzene rings [centroid–centroid distance = 3.862 (1) Å].

## Related literature

For general background to oxime compounds, see: Chaudhuri (2003 ▶); Dong *et al.* (2007*a* ▶, 2008 ▶). For related structures, see: Dong *et al.* (2007*b* ▶, 2009 ▶). For the ligand synthesis, see: Wang *et al.* (2008 ▶); Zhao *et al.* (2009 ▶).
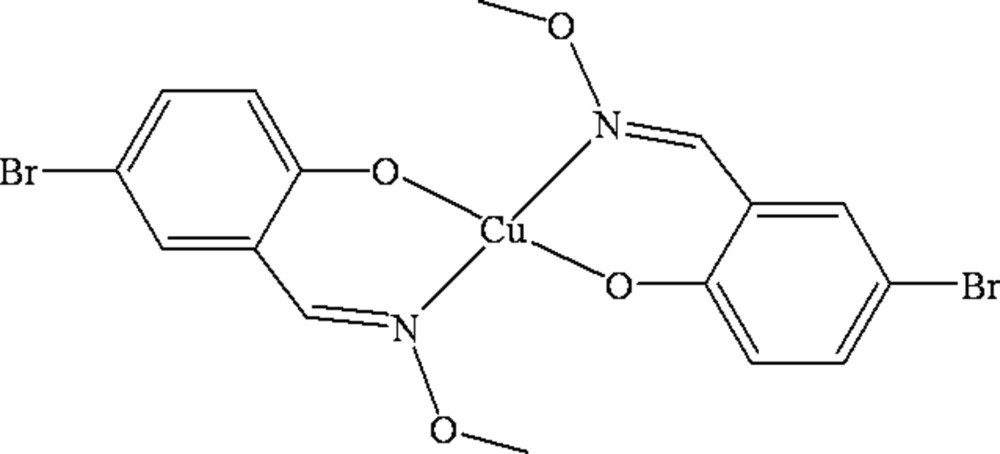


## Experimental

### 

#### Crystal data

[Cu(C_8_H_7_BrNO_2_)_2_]
*M* *_r_* = 521.65
Monoclinic, 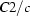

*a* = 24.691 (3) Å
*b* = 3.8623 (5) Å
*c* = 20.260 (2) Å
β = 117.453 (2)°
*V* = 1714.4 (3) Å^3^
*Z* = 4
Mo *K*α radiation
μ = 5.96 mm^−1^
*T* = 298 K
0.40 × 0.12 × 0.11 mm

#### Data collection

Siemens SMART 1000 CCD diffractometer
Absorption correction: multi-scan (*SADABS*; Sheldrick, 1996 ▶) *T*_min_ = 0.199, *T*_max_ = 0.560
3981 measured reflections
1521 independent reflections
1128 reflections with *I* > 2σ(*I*)
*R*_int_ = 0.040

#### Refinement

*R*[*F* ^2^ > 2σ(*F* ^2^)] = 0.032
*wR*(*F* ^2^) = 0.053
*S* = 1.04
1521 reflections
116 parameters
H-atom parameters constrained
Δρ_max_ = 0.54 e Å^−3^
Δρ_min_ = −0.40 e Å^−3^

### 

Data collection: *SMART* (Siemens, 1996 ▶); cell refinement: *SAINT* (Siemens, 1996 ▶); data reduction: *SAINT*; program(s) used to solve structure: *SHELXS97* (Sheldrick, 2008 ▶); program(s) used to refine structure: *SHELXL97* (Sheldrick, 2008 ▶); molecular graphics: *SHELXTL* (Sheldrick, 2008 ▶) and *Mercury* (Macrae *et al.*, 2006 ▶); software used to prepare material for publication: *SHELXTL*.

## Supplementary Material

Crystal structure: contains datablocks global, I. DOI: 10.1107/S1600536809047989/hy2253sup1.cif

Structure factors: contains datablocks I. DOI: 10.1107/S1600536809047989/hy2253Isup2.hkl

Additional supplementary materials:  crystallographic information; 3D view; checkCIF report

## Figures and Tables

**Table 1:** Selected bond lengths (Å)

Cu1—O2	1.910 (2)
Cu1—N1	2.000 (3)

**Table 2:** Hydrogen-bond geometry (Å, °)

*D*—H⋯*A*	*D*—H	H⋯*A*	*D*⋯*A*	*D*—H⋯*A*
C1—H1*C*⋯O1^i^	0.96	2.52	3.328 (5)	142

Symmetry code: (i) 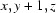.

**Fig. 1.**
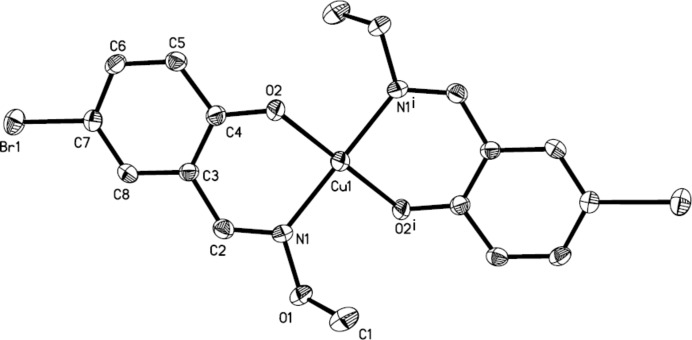
The molecular structure of the title compound. Displacement ellipsoids are drawn at the 30% probability level. H atoms have been omitted. [Symmetry code: (i) 1-x, 1-y, 1-z.]

**Fig. 2.**
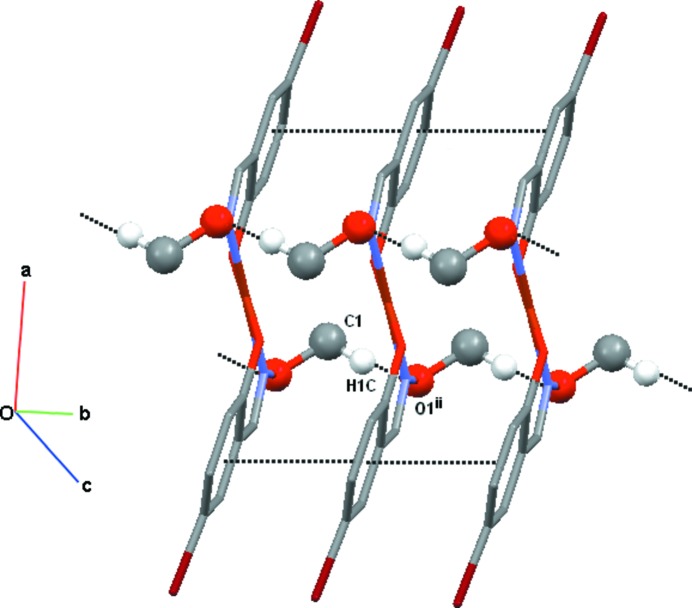
Packing diagram for the title compound, showing the one-dimensional supramolecular structure formed by intermolecular C—H···O hydrogen bonds (dashed lines) and π–π stacking interactions (dashed lines). H atoms not involved in hydrogen bonding have been omitted for clarity. [Symmetry code: (ii) x, 1+y, z.]

## supplementary crystallographic information

### Comment

Oximes are a traditional class of chelating ligands widely used in coordination and analytical chemistry and extraction metallurgy (Chaudhuri, 2003; Dong *et al.*, 2007a,b, 2008, 2009). We report here the title mononuclear copper(II) complex with an oxime-type ligand.

The molecular structure of the title compound is shown in Fig. 1. The Cu^II^ ion, lying on an inversion centre is four-coordinated in a *trans*-CuN_2_O_2_ square-planar geometry, with two phenolate O atoms and two oxime N atoms from two N,O-bidentate oxime-type ligands. Bond lengths and angles are within normal ranges (Table 1). The Cu—O and Cu—N bond lengths are 1.910 (2) Å and 2.000 (3) Å, respectively, which are slightly longer than those observed in a similar Schiff base copper(II) complex [the mean bond lengths of Cu—O and Cu—N are 1.894 (2) and 1.990 (3) Å] (Dong *et al.*, 2009).

In the crystal structure, intermolecular C1—H1C···O1 hydrogen bonds link neighbouring molecules into a one-dimensional supramolecular structure, with an *R*_2_^2^(14) ring motif (Table 2 and Fig. 2). The one-dimensional structure is further stabilized by weak π–π stacking interactions between the adjacent benzene rings [centroid–centroid distance = 3.862 (1) Å] (Fig. 2).

### Experimental

(*E*)-5-Bromo-2-hydroxybenzaldehyde *O*-methyl oxime (H*L*) was synthesized according to an analogous method in literature (Wang *et al.*, 2008; Zhao *et al.*, 2009). A blue solution of copper(II) acetate monohydrate (1.7 mg, 0.008 mmol) in methanol (4 ml) was added dropwise to a solution of H*L* (4.1 mg, 0.016 mmol) in methanol (5 ml) at room temperature. The colour of the mixing solution turned to yellow immediately then turned to brown slowly. The mixture was allowed to stand at room temperature for several days. With evaporating of the solvent, dark-brown needle-like single crystals suitable for X-ray crystallographic analysis were obtained (yield 49.3%). IR: ν(C═N) 1607, ν(Ar—O) 1243, ν(Cu—N) 447, ν(Cu—O) 422 cm^-1^. Analysis, calculated for C_16_H_14_Br_2_CuN_2_O_4_: C 39.30, H 3.32, Cu 11.51, N 5.13%; found: C 39.21, H 3.39, Cu 11.64, N 4.85%.

### Refinement

H atoms were positioned geometrically and refined as riding atoms, with C—H = 0.96 (CH_3_) and 0.93 Å (CH) and with *U*_iso_(H) = 1.2(1.5 for methyl)*U*_eq_(C).

### Crystal data

**Table:**

[Cu(C_8_H_7_BrNO_2_)_2_]	*F*(000) = 1020
*M**_r_* = 521.65	*D*_x_ = 2.021 Mg m^−^^3^
Monoclinic, *C*2/*c*	Mo *K*α radiation, λ = 0.71073 Å
Hall symbol: -C 2yc	Cell parameters from 1233 reflections
*a* = 24.691 (3) Å	θ = 2.2–23.4°
*b* = 3.8623 (5) Å	µ = 5.96 mm^−^^1^
*c* = 20.260 (2) Å	*T* = 298 K
β = 117.453 (2)°	Needle-like, dark-brown
*V* = 1714.4 (3) Å^3^	0.40 × 0.12 × 0.11 mm
*Z* = 4	

### Data collection

**Table:**

Siemens SMART 1000 CCD diffractometer	1521 independent reflections
Radiation source: fine-focus sealed tube	1128 reflections with *I* > 2σ(*I*)
graphite	*R*_int_ = 0.040
φ and ω scans	θ_max_ = 25.0°, θ_min_ = 1.9°
Absorption correction: multi-scan (*SADABS*; Sheldrick, 1996)	*h* = −21→28
*T*_min_ = 0.199, *T*_max_ = 0.560	*k* = −4→4
3981 measured reflections	*l* = −24→23

### Refinement

**Table:**

Refinement on *F*^2^	Primary atom site location: structure-invariant direct methods
Least-squares matrix: full	Secondary atom site location: difference Fourier map
*R*[*F*^2^ > 2σ(*F*^2^)] = 0.032	Hydrogen site location: inferred from neighbouring sites
*wR*(*F*^2^) = 0.053	H-atom parameters constrained
*S* = 1.03	*w* = 1/[σ^2^(*F*_o_^2^) + (0.0126*P*)^2^] where *P* = (*F*_o_^2^ + 2*F*_c_^2^)/3
1521 reflections	(Δ/σ)_max_ < 0.001
116 parameters	Δρ_max_ = 0.54 e Å^−^^3^
0 restraints	Δρ_min_ = −0.40 e Å^−^^3^

### Fractional atomic coordinates and isotropic or equivalent isotropic displacement parameters (Å^2^)

**Table:**

	*x*	*y*	*z*	*U*_iso_*/*U*_eq_	
Cu1	0.5000	0.5000	0.5000	0.0478 (2)	
Br1	0.169435 (18)	0.60817 (11)	0.35138 (2)	0.04703 (16)	
N1	0.46014 (14)	0.4521 (8)	0.56593 (15)	0.0359 (8)	
O1	0.48859 (12)	0.2994 (7)	0.63799 (13)	0.0454 (7)	
O2	0.43631 (11)	0.8108 (7)	0.43906 (13)	0.0459 (8)	
C1	0.54119 (19)	0.4966 (11)	0.6868 (2)	0.0563 (13)	
H1A	0.5672	0.5306	0.6638	0.084*	
H1B	0.5632	0.3737	0.7328	0.084*	
H1C	0.5284	0.7173	0.6964	0.084*	
C2	0.40218 (17)	0.4722 (9)	0.54291 (19)	0.0353 (10)	
H2	0.3870	0.3956	0.5747	0.042*	
C3	0.35910 (16)	0.6052 (9)	0.47123 (18)	0.0303 (9)	
C4	0.37870 (17)	0.7699 (10)	0.42340 (19)	0.0337 (10)	
C5	0.33223 (17)	0.9004 (10)	0.35507 (19)	0.0357 (10)	
H5	0.3434	1.0184	0.3232	0.043*	
C6	0.27127 (17)	0.8574 (9)	0.33473 (19)	0.0358 (10)	
H6	0.2418	0.9423	0.2894	0.043*	
C7	0.25404 (16)	0.6861 (9)	0.3825 (2)	0.0308 (9)	
C8	0.29692 (16)	0.5679 (9)	0.45035 (19)	0.0323 (9)	
H8	0.2847	0.4628	0.4826	0.039*	

### Atomic displacement parameters (Å^2^)

**Table:**

	*U*^11^	*U*^22^	*U*^33^	*U*^12^	*U*^13^	*U*^23^
Cu1	0.0365 (4)	0.0708 (6)	0.0413 (4)	0.0207 (4)	0.0224 (4)	0.0218 (4)
Br1	0.0330 (3)	0.0476 (3)	0.0530 (3)	−0.0031 (2)	0.0134 (2)	−0.0005 (2)
N1	0.035 (2)	0.044 (2)	0.0273 (18)	0.0067 (17)	0.0131 (15)	0.0089 (15)
O1	0.0378 (17)	0.057 (2)	0.0334 (15)	0.0074 (14)	0.0095 (13)	0.0140 (14)
O2	0.0311 (17)	0.064 (2)	0.0454 (16)	0.0148 (15)	0.0198 (13)	0.0245 (14)
C1	0.057 (3)	0.060 (3)	0.037 (2)	0.003 (2)	0.009 (2)	−0.001 (2)
C2	0.038 (3)	0.036 (3)	0.036 (2)	0.001 (2)	0.020 (2)	0.0039 (18)
C3	0.034 (2)	0.032 (2)	0.026 (2)	0.0051 (19)	0.0140 (18)	0.0016 (19)
C4	0.034 (2)	0.037 (2)	0.030 (2)	0.007 (2)	0.015 (2)	0.0004 (19)
C5	0.043 (3)	0.036 (2)	0.033 (2)	0.008 (2)	0.0216 (19)	0.004 (2)
C6	0.036 (3)	0.038 (3)	0.027 (2)	0.009 (2)	0.0097 (19)	0.003 (2)
C7	0.031 (2)	0.024 (2)	0.036 (2)	0.0008 (18)	0.0148 (19)	−0.0039 (18)
C8	0.039 (2)	0.032 (2)	0.034 (2)	0.000 (2)	0.0224 (19)	0.0001 (19)

### Geometric parameters (Å, °)

**Table:**

Cu1—O2	1.910 (2)	C2—H2	0.9300
Cu1—N1	2.000 (3)	C3—C8	1.399 (5)
Br1—C7	1.907 (4)	C3—C4	1.418 (5)
N1—C2	1.287 (4)	C4—C5	1.422 (5)
N1—O1	1.424 (3)	C5—C6	1.375 (5)
O1—C1	1.435 (4)	C5—H5	0.9300
O2—C4	1.316 (4)	C6—C7	1.391 (5)
C1—H1A	0.9600	C6—H6	0.9300
C1—H1B	0.9600	C7—C8	1.370 (5)
C1—H1C	0.9600	C8—H8	0.9300
C2—C3	1.442 (5)		
			
O2^i^—Cu1—O2	180.000 (2)	C3—C2—H2	117.7
O2^i^—Cu1—N1	91.27 (11)	C8—C3—C4	120.8 (3)
O2—Cu1—N1	88.73 (11)	C8—C3—C2	117.7 (3)
O2^i^—Cu1—N1^i^	88.73 (11)	C4—C3—C2	121.5 (3)
O2—Cu1—N1^i^	91.27 (11)	O2—C4—C3	124.1 (3)
N1—Cu1—N1^i^	180.000 (1)	O2—C4—C5	119.3 (3)
C2—N1—O1	109.7 (3)	C3—C4—C5	116.6 (3)
C2—N1—Cu1	124.0 (2)	C6—C5—C4	122.0 (4)
O1—N1—Cu1	124.1 (2)	C6—C5—H5	119.0
N1—O1—C1	110.4 (3)	C4—C5—H5	119.0
C4—O2—Cu1	123.8 (2)	C5—C6—C7	119.5 (3)
O1—C1—H1A	109.5	C5—C6—H6	120.2
O1—C1—H1B	109.5	C7—C6—H6	120.2
H1A—C1—H1B	109.5	C8—C7—C6	120.9 (4)
O1—C1—H1C	109.5	C8—C7—Br1	120.0 (3)
H1A—C1—H1C	109.5	C6—C7—Br1	119.0 (3)
H1B—C1—H1C	109.5	C7—C8—C3	120.1 (3)
N1—C2—C3	124.5 (3)	C7—C8—H8	120.0
N1—C2—H2	117.7	C3—C8—H8	120.0
			
O2^i^—Cu1—N1—C2	−148.5 (3)	C8—C3—C4—O2	−179.3 (3)
O2—Cu1—N1—C2	31.5 (3)	C2—C3—C4—O2	1.1 (6)
O2^i^—Cu1—N1—O1	13.2 (3)	C8—C3—C4—C5	1.5 (5)
O2—Cu1—N1—O1	−166.8 (3)	C2—C3—C4—C5	−178.1 (3)
C2—N1—O1—C1	−133.0 (3)	O2—C4—C5—C6	178.2 (3)
Cu1—N1—O1—C1	63.0 (3)	C3—C4—C5—C6	−2.6 (5)
N1—Cu1—O2—C4	−38.8 (3)	C4—C5—C6—C7	1.0 (6)
N1^i^—Cu1—O2—C4	141.2 (3)	C5—C6—C7—C8	1.8 (5)
O1—N1—C2—C3	−178.3 (3)	C5—C6—C7—Br1	−176.9 (3)
Cu1—N1—C2—C3	−14.3 (5)	C6—C7—C8—C3	−2.8 (5)
N1—C2—C3—C8	171.7 (3)	Br1—C7—C8—C3	175.9 (3)
N1—C2—C3—C4	−8.6 (6)	C4—C3—C8—C7	1.1 (5)
Cu1—O2—C4—C3	29.8 (5)	C2—C3—C8—C7	−179.2 (3)
Cu1—O2—C4—C5	−151.0 (3)		

Symmetry codes: (i) −*x*+1, −*y*+1, −*z*+1.

### Hydrogen-bond geometry (Å, °)

**Table:**

*D*—H···*A*	*D*—H	H···*A*	*D*···*A*	*D*—H···*A*
C1—H1C···O1^ii^	0.96	2.52	3.328 (5)	142

Symmetry codes: (ii) *x*, *y*+1, *z*.
